# Microbiome-host-immune crosstalk: mining the microbiome: a treasure trove waiting to be unlocked

**DOI:** 10.1038/s41435-021-00148-2

**Published:** 2021-07-27

**Authors:** Mario Noti, Thomas Brunner

**Affiliations:** 1 Nestlé SA, Nestlé Research, Nestlé Institute of Health Sciences, Department of Gastrointestinal Health Immunology, Vers-Chez-les-Blancs, CH-1000 Lausanne, Switzerland; 2 Biochemical Pharmacology, Department of Biology, University of Konstanz, Konstanz, Germany

**Keywords:** Mucosal immunology, Inflammation

During the last century, mankind launched a war against microbes and medicine scored big with the discovery of antimicrobials. These treatments saved and continue saving millions of lives every year. However, at the time we developed these armed forces we did not have the understanding how important the microbiome is to our health, and how these new weapons harm our own lines of defense. With the trillions of microbes that call us home, we form a strong coalition that few enemies dare to fight. However, given successes of modern medicine, we forgot to care for our most intimate friends. And the recent pandemic has further fueled germophobic habits associated with modern life. Besides frequent antibiotic exposure, poor dietary choices take a toll on our microbes that protect us from invasion of pathogens and provide us with vitamins and metabolites that are key to sustain health. With a growing understanding of the importance of the microbiome for immune maturation and organismal health it is time to end the relentless war on our microbes. We may have lost some of our intimate friends in this battle already, but it is not too late to reinforce this alliance to regain to old strength.

Understanding complex microbial ecosystems such as the human gut microbiome requires information about both microbial species and the metabolites they produce. These metabolites are exchanged via a large network of cross-feeding interactions. While technological advances make it possible to gain information about these networks, how the microbial communicome affects host physiology adds yet another layer of complexity. With the renaissance of microbiome work due to technological advances, we started to scratch the tip of the iceberg to unlock some of the secrets of our intimate friends that we can leverage to support health and wellbeing.

In this special issue in *Genes & Immunity*, we have attempted to provide a snapshot of current research activities aiming to gain a better understanding of the co-evolved, bi-directional crosstalk between the microbiome and the host immune system. We thank our colleagues that contributed to this section on microbiome-host-immune crosstalk, and we hope you will enjoy reading and pondering over the secrets and treasures that our intimate friends hold. Mining the microbiome—a treasure trove waiting to be unlocked or to be more pragmatic—from symbiosis to therapy.

The co-existence of immune cells and trillions of beneficial commensal microorganisms in the gastrointestinal tract requires tightly controlled barrier and regulatory mechanisms to sustain tissue homeostasis. This homeostasis is regulated through diverse functions of intestinal epithelial cells (IECs) that—uniquely equipped like a Swiss army knife—not only provide a physical- and biochemical barrier but also allow for microbial sensing to direct appropriate downstream immune signaling. The review by Eshleman and Alenghat provides a comprehensive overview of how IECs communicate with our intimate friends and the necessity of this crosstalk to maintain immune cell homeostasis in the intestine.

Mix and colleagues further elaborate on the IEC-microbe crosstalk. Adhesins are critical for the colonization of surface epithelia by bacteria via interaction with specific host cell receptors. Successful colonization of epithelia is an important infectious disease-promoting parameter, and bacteria-elicited cellular responses of surface epithelia preventing this microbial adhesion and colonization contribute to the host defense. In their review, Mix and colleagues discuss how bacteria and host epithelial cells interact and communicate with each other on a biochemical, chemical, and microscale level, and how this crosstalk determines the success of epithelial surface colonization.

The discovery of antimicrobials is probably one of the most successful stories in the history of medicine. While antibiotics are not only efficient in the eradication of harmful bacteria, they also affect the resident microbiota that naturally provides one of our most effective barriers against infection by pathogenic microbes. Although most bacterial groups recover after antibiotic treatment, some taxa fail to do so resulting in altered microbial community structures and opening of niches for pathogenic microbes that can carry antibiotic resistance genes. The review by Panwar and colleagues discusses the natural barriers of pathogen resistance and the potential to leverage the vast potential of the microbiota as an alternative means to combat infection by antibiotic-resistant pathogens.

The intestinal microbiome is not only a rich source of different bacterial strains and associated genes, but also of a complex library of carbohydrates, generated of differentially interlinked sugar molecules. Microbial carbohydrates not only trigger immune responses in our body but help also to select and shape the carbohydrate-specific antibody repertoire. Kappler and Hennet discuss how structural similarities of carbohydrates in intestinal bacteria, and protist, fungi, and animals result in the production of protective antibody responses, and on the downside how mimicry between bacterial and host glycoconjugates causes antibody cross-reactivity and autoimmune diseases.

Schär and Hapfelmeier summarize current knowledge of bacterial immune recognition at mucosal sites with a special focus on Th17 immune responses. Given that pathobionts, pathogens, or bacterial toxins can trigger Th17-mediated mucosal immunity, identification of adjuvants for inducing robust non-pathogenic Th17 responses may foster the design of effective mucosal vaccines urgently needed to reduce the global burden of infectious disease. Once more our little friends—some of which known to induce nonpathogenic Th17 responses—may teach us important lessons to overcome the lack of effective mucosal vaccine delivery systems.

Unlike the gut that provides an almost ideal habitat for the growth of bacteria, the skin is a hostile place for microbes given its dry, acidic, and nutrient-poor characteristics. Nevertheless, a diverse array of microbes that include bacteria, viruses, fungi, and archae have co-evolved with these harsh environmental conditions and call the skin their home. Lunjani and colleagues highlight recent advances in our understanding of skin-microbiome-immune interactions and how this knowledge can be further leveraged for targeted and personalized microbial solutions to effectively treat or prevent skin disorders.

Like aging host cellular systems, the gut microbiome undergoes significant changes through time as it integrates and responds to signals from the environment. However, our knowledge of bacterial evolution and associated functional consequences for host fitness remain enigmatic. Last but not least, the review by Bosco and Noti gives an overview on hallmarks of the aging immune system and gut microbiome, how age-related changes in commensal community structures impact host immunity and discuss emerging strategies to reinforce age-related declines in immune cell functioning through microbiome modulation or rejuvenation.

